# Effect of walnut leaves on oxidative stress caused by murine cerebral malaria

**DOI:** 10.3389/fcimb.2025.1636404

**Published:** 2025-09-10

**Authors:** Rewaida Abdel-Gaber, Afra Alharbi, Nada Almohawis, Saleh Al Quraishy, Esam Al-Shaebi

**Affiliations:** ^1^ Department of Zoology, College of Science, King Saud University, Riyadh, Saudi Arabia; ^2^ Department of Chemistry, College of Science, King Saud University, Riyadh, Saudi Arabia

**Keywords:** model mice, natural products, oxidative stress, *Plasmodium berghei*, protein expression

## Abstract

**Background:**

Following the infection of mice by the *Plasmodium* parasite, a significant increase in oxidative stress occurs within the brain. This oxidative stress is further intensified as the parasite proliferates, leading to an imbalance in the body’s oxidant and antioxidant systems. As a result, the affected mice experience various health issues stemming from this disruption. Previous research has indicated that the leaves of *Juglans regia*, commonly known as walnut, possess protective properties that can mitigate brain damage caused by the *Plasmodium* parasite. These leaves inhibit the parasite’s reproduction and restore normal brain functions in the affected mice.

**Purpose:**

In the current study, we investigated the impact of *J. regia* leaves on oxidative stress and cellular damage associated with cerebral malaria infection in a murine model.

**Methods:**

The extract of leaves from *Juglans regia* was prepared using methanol as the solvent. Thirty female C57BL/6 mice, weighing 20 to 25 grams and aged 9 to 12 weeks, were organized into six distinct groups for the experiment (labeled G1 through G6). On day 9, following the initiation of the infection protocol, all mice were euthanized, and their brains were harvested for further analysis. The primary focus of this study was to assess the degree of oxidative stress present in the brain tissue and measure the activities of various antioxidant enzymes. To quantify levels of inducible nitric oxide synthase (iNOS), the Enzyme-Linked Immunosorbent Assay (ELISA) technique and immunohistochemistry assay were employed, providing a sensitive and specific means of detecting this enzyme’s concentration in the brain tissue samples.

**Results:**

The study findings revealed that the heightened levels of free radicals in the brain, induced by the infection with *Plasmodium berghei*, were effectively eliminated following a daily treatment regimen with JRLE. This treatment resulted in notable reductions in the concentration of key oxidative stress markers, including nitric oxide (NO), malondialdehyde (MDA), and hydrogen peroxide (H_2_O_2_), in the groups of mice that received JRLE compared to those that remained infected. Moreover, the administration of JRLE appeared to play a protective role against oxidative stress by enhancing the activities of several crucial antioxidant enzymes. Specifically, there was a marked increase in the activity levels of catalase (CAT), superoxide dismutase (SOD), reduced glutathione (GSH), and total antioxidant capacity (TAC) in the treated groups. Interestingly, although the treatment significantly increased the expression levels of inducible nitric oxide synthase (iNOS), the subsequent administration of JRLE effectively mitigated this increase.

**Conclusion:**

This comprehensive evaluation aimed to clarify the potential protective effects of *J. regia* leaf extract concerning oxidative stress and its related neurological implications induced by *P. berghei* infection. Therefore, these plant leaves are an alternative source of new antioxidants and antimalarial agents.

## Introduction

Malaria is a serious and often life-threatening infectious disease caused by protozoan parasites from the genus *Plasmodium*. There are four main species known to infect humans: *Plasmodium falciparum*, *Plasmodium vivax*, *Plasmodium ovale*, and *Plasmodium malariae*. Malaria usually involves the production of large amounts of reactive oxygen species (ROS) by both the host and parasite, leading to oxidative and pathological processes in the host system most of the time (Akanbi et al., 2009). Among these species, *P. falciparum* is generally considered the most virulent and pathogenic, responsible for the most severe cases and deaths worldwide (Marteau et al., 2021). This species can cause complications such as cerebral malaria (CM), severe anemia, and multi-organ failure, making it a major public health concern, especially in tropical and subtropical regions where the disease is endemic. CM is a serious complication resulting from *P. falciparum* infection in humans (Penet et al., 2005). The murine model using *Plasmodium berghei* has been widely employed to understand the process leading to CM (Martins et al., 2016; Al-Shaebi et al., 2017; Alharbi et al., 2025), but there is significant debate over the usefulness of these models and whether their study applies to human disease (de Souza et al., 2010).

Most antimalarial drugs, including well-known medications like chloroquine, artemisinin-based combination therapies (ACTs), mefloquine, and atovaquone/proguanil, work by disrupting the normal growth, metabolism, and reproductive processes of the malaria-causing parasite *Plasmodium* (Olanrewaju and Johnson, 2001; Percário et al., 2012). These drugs are designed to target specific stages of the parasite’s lifecycle, effectively reducing its viability within the human host. However, the extensive and often indiscriminate use of these antimalarial agents has led to a significant problem: the emergence of drug resistance. This resistance occurs when the parasites adapt to the medications, making them less effective or completely ineffective over time, which makes malaria harder to treat and control (Olayemi et al., 2012). Understanding the biology and transmission patterns of the *Plasmodium* parasite is crucial for developing effective prevention and treatment methods. Herbal plants and their bioactive phytochemicals offer several benefits, including minimal drug residues and side effects, a reduced risk of developing drug resistance, and lower costs. Therefore, they are considered promising candidates for anti-malarial treatments (Enechi et al., 2019).

*Juglans regia*, commonly known as the English walnut, has long been used in traditional medicine for its therapeutic benefits. Numerous studies have demonstrated the antioxidant (McKay et al., 2010), anticancer (Soto-Maldonado et al., 2019), anti-inflammatory (Igbayilola et al., 2022), anti-diabetic (Elouafy et al., 2023a), and antimicrobial (Elouafy et al., 2023b) properties of *J. regia*. Recent studies have demonstrated its efficacy as a potential treatment for malaria and in providing neuroprotective benefits. According to the study by Alharbi et al. (2025), extracts from *J. regia* can significantly preserve brain health in mouse models and limit the growth of malaria parasites. The phytochemical components of the plant that have already been identified may be the cause of these reported actions (Aja et al., 2017). This illustrates the value of exploring natural compounds for infectious disease treatment and neurological protection.

In order to elucidate its protective mechanism, this study examined the impact of the methanolic extract derived from *J. regia* leaves on the oxidative damage caused by the *Plasmodium berghei* parasite.

## Materials and methods

### Plant collection and reference drug

Fresh leaves of the walnut tree, scientifically known as *Juglans regia*, were gathered for this investigation from the study area of Al Bahah City (Saudi Arabia). Each plant specimen was carefully verified at the herbarium of the Botany Department at King Saud University, ensuring precise identification and classification. To ensure appropriate documentation and support future research, these specimens were given a special voucher number, KSU-21595. For comparative examination in the study, chloroquine diphosphate salt—an established and widely recognized antimalarial medication—was sourced from Sigma-Aldrich (St. Louis, USA), serving as a standard antimalarial reference to evaluate the properties of the walnut leaves.

### Preparation of *Juglans regia* leaf extracts

At room temperature, the walnut leaves were allowed to undergo a natural air-drying process, allowing moisture to evaporate gradually and preserving their chemical composition. Once thoroughly dried, the leaves were ground into a fine powder using a Hummer Grinder (Edison Electric, ED-CG1400, China), ensuring a uniform consistency. Approximately 100 grams of this powdered sample was placed in a container where it was gently agitated while being immersed in 1,000 milliliters of 70% methanol. This percolation process lasted for a full 24 hours at room temperature, facilitating the extraction of soluble compounds from the leaves. After the extraction period, the mixture was carefully strained using Whatman filter paper No. 1, which effectively separated the liquid extract from the solid leaf residue. The resulting filtrate was then subjected to a rotary vacuum evaporator, specifically a Buchi model from Switzerland, where it was dried at a controlled temperature of 45 °C. This process concentrated the extract, leaving behind a potent methanolic crude extract, which was subsequently diluted with distilled water in a weight-to-volume ratio to prepare the desired dosage for further use.

### Gas chromatography-mass spectrometry analysis

A precise volume of 1 µL was injected into the system using the advanced autosampler injection mechanism of the Agilent Technologies GC-MS 7890B Gas Chromatography-Mass Spectrometry system, based in Santa Clara, CA, USA. The components of the sample were expertly identified with the assistance of sophisticated database-integrated software, specifically the NIST-MS database. The analysis was conducted using Gas Chromatography combined with a mass-selective detector (GC-MS), enabling detailed separation and identification of the target compounds. For this process, a DB-5 MS capillary column from Agilent Technologies was employed. This column featured an impressive length of 30 meters, a narrow internal diameter of 0.25 mm, and a phase thickness also measuring 0.25 mm, designed to optimize separation efficiency. Helium was utilized as the carrier gas, flowing at a steady rate of 1 ml/min. The inlet temperature was precisely maintained at 250 °C, operating under a split mode ratio of 50. In the oven, the temperature was systematically varied between 50 °C and 250 °C, culminating in a comprehensive analysis that lasted a total of 61 min. The settings for the mass spectrometer (MS) detector were carefully configured to maximize data collection. This included an acquisition scan type covering a wide mass range from 40 to 500 g/mol, a scan speed adjusted to 1.56, and a solvent delay strategically set at 4 min. The temperature of the MS source was rigorously controlled at 230°C, ensuring optimal conditions for the detection and identification of the sample components throughout the analysis.

### *Plasmodium* parasite activation

The cryopreserved *Plasmodium* (Pb) parasite was carefully passaged four times in laboratory mice of the species *Mus musculus*, using a sample size of five animals. To quantify the level of infection, referred to as parasitemia, thin blood smears obtained from the tails of the infected mice were stained with a 10% Giemsa solution (Sigma-Aldrich). This staining process followed the methodology established by Hilou et al. (2006). Assessment of parasitemia, expressed as the percentage of parasitized red blood cells (pRBCs), was conducted through microscopic enumeration using a Neubauer Hemocytometer, utilizing the formula: [(number of pRBCs)/(total number of RBCs counted)]×100. When the parasitemia reached approximately 20%, blood containing pRBCs was carefully collected in a heparinized tube to prevent clotting and was then diluted with 5 ml of PBS, maintaining a pH of 7.4. Subsequently, a precise volume of 0.2 ml, containing 1×10^5^ of the *Pb* parasites, was administered to each female C57BL/6 mouse utilizing the intraperitoneal injection method.

### Animals and experimental design

**Figure 1:**
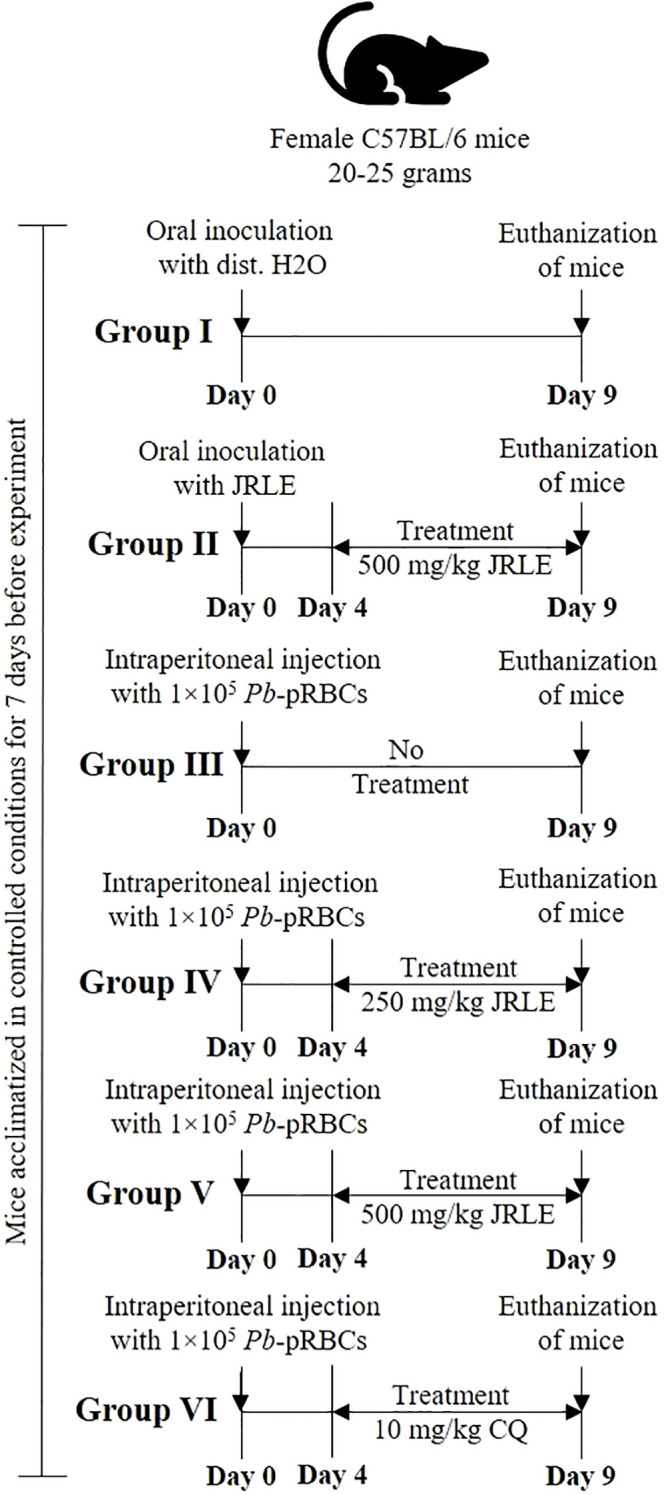
Experimental flowchart detailing the treatment of female C57BL/6 mice, weighing 20-25 grams, over nine days. Group I receives oral distilled water; Group II receives oral JRLE starting from Day 4; Group III receives an intraperitoneal injection of 1×10⁵ Pb-pRBCs with no treatment; Group IV receives the same injection and treatment with 250 mg/kg JRLE starting from Day 4; Group V also receives the injection and 500 mg/kg JRLE starting from Day 4; Group VI also receives the injection and treated with 10 mg/kg CQ starting from Day 4. All groups undergo euthanization on Day 9.

In this study, we acquired 30 female C57BL/6 mice, each weighing between 20 and 25 grams and aged between 9 and 12 weeks, from the College of Pharmacy’s animal facility at King Saud University in Saudi Arabia. Before their experimental use, the mice were housed for 7 days in five groups within polypropylene cages, allowing them time to acclimate to their new environment. These mice were kept under controlled conditions, with a 12-hour light/dark cycle maintained at 23 ± 2°C. They had unrestricted access to water and were provided with a standard laboratory animal diet to ensure their well-being. All research procedures were approved by the King Saud University Research Ethics Committee (REC) for Laboratory Animal Care, under the reference KSU-SE-24-74, ensuring adherence to ethical standards. The experimental mice were systematically divided into six groups, each consisting of five individuals, as illustrated in **Figure 1**. On day 4 following infection, treatments were administered orally as follows: groups (4 and 5) were orally treated with different concentrations of JRLE (250 and 500 mg/kg, respectively), according to the previous study of Sharif et al. (2022) and Alharbi et al. (2025), and group (6) was treated with chloroquine, according to Abay et al. (2015) and Al-Shaebi et al. (2017). On the ninth day following infection, the level of parasitemia in each infected experimental mouse was assessed using a thin blood smear technique, which had been previously described in the study. The percentage of parasitemia was calculated according to the established methodology, allowing for a precise evaluation of the infection’s progression. Neurological symptoms were assessed to evaluate the onset of the disease. The diagnosis of CM was based on clinical signs, such as paralysis and ataxia.

### Sample collection

On the final day of the experiment, designated as day 9, the mice were euthanized humanely following ethical standards. Following this, a dissection process was conducted to collect the brain samples. These samples were preserved using several methods to facilitate a range of future analyses. Firstly, some brain tissues were immersed in neutral buffered formalin (NBF), a fixative commonly used for immunohistochemistry studies, to ensure the preservation of cellular structures and proteins for detailed microscopic examination. Secondly, additional samples were placed in small tubes and stored at -80°C. This step was taken to maintain their integrity for subsequent investigations into oxidative status, allowing for the assessment of any oxidative damage or stress markers. Lastly, brain samples were also preserved in RNA later, a stabilization solution that maintains RNA integrity, and these samples were stored at -80°C for protein expression analysis, enabling a comprehensive exploration of gene activity within the brain post-experiment.

### Biochemical analysis

Brain tissue was carefully homogenized to a concentration of 10% (w/v) using ice-cold 0.1 M phosphate buffer, adjusted to a pH of 7.4. This process was essential for preserving the integrity of the samples and ensuring accurate measurements of oxidative stress parameters. Following homogenization, the mixture was subjected to centrifugation at 3000 rpm for 15 min at a temperature of 4 °C. This step allowed for the separation of cellular debris, resulting in a supernatant that was then stored at -20 °C for future analysis. In the subsequent analyses, oxidative markers were measured in the supernatant of the brain homogenate using specific diagnostic kits obtained from Bio-Diagnostic Co. (Egypt). Various parameters were assessed, including Catalase (CAT) following the method described by Aebi (1984), Glutathione reduced (GSH) based on protocol of Ellman (1959), Nitric Oxide (NO) as per the technique outlined by Green et al. (1982), Hydrogen Peroxide (H_2_O_2_) again referencing Aebi (1984) work, Malondialdehyde (MDA) according to the method established by Ohkawa et al. (1979), Total Antioxidant Capacity (TAC) following Koracevic et al. (2001) approach, and Superoxide Dismutase (SOD) utilizing Nishikimi et al. (1972) method. The absorbance of the samples was precisely measured using the Spectra MAX 190, with the analysis supported by SoftMax^®^ Pro software version 6.3.1, ensuring reliable and accurate quantitative assessments. This approach allowed for a thorough investigation of oxidative stress within the brain tissue samples.

### Immunohistochemical staining of Inducible nitric oxide synthase

The fixed brain tissues were dehydrated in graded ethyl alcohol, treated with xylene, soaked in paraffin wax, and finally cut into 5 µm sections using a microtome machine. The sections processed for immunohistochemical labeling were deparaffinized in xylene and rehydrated, and endogenous peroxidase activity was extinguished through incubation with 3% H_2_O_2_ for 5 min. Sections were then pre-incubated for 30 min with normal serum buffer solution (Diagnostic BioSystems, Serpentine, CA, USA), and incubated for 3 hr at 4 °C with 1:300 dilution of anti-iNOs antibodies (Santa Cruz Biotechnology, CA, USA), followed by a biotinylated secondary antibody and streptavidin-conjugated horseradish peroxidase (Vision Biosystems Novocastra, Novocastra Laboratories Ltd., Newcastle, UK) prepared according to the kit instructions. The sections were incubated with 3,3’-diaminobenzidine hydrochloride (DAB) chromogen substrate (Vision Biosystems Novocastra) according to the manufacturer’s instructions and counterstained with H&E (Sigma Chemical Co.). Thorough washes between steps were performed using an immune wash buffer (Vision Biosystems Novocastra). Sections were dehydrated through a graded ethanol series, cleared in xylene, and covered with a thin glass coverslip. All sections were examined for the apoptotic marker of iNOs and photographed using an Olympus B×61 microscope (Tokyo, Japan). The immunostaining intensity was scored from 0-3 (0-negative staining, 1-mild, 2-moderate, and 3-strong immunostaining), according to Punsawad et al. (2013).

### Sandwich enzyme-linked immunosorbent assay

To assess the levels of inducible nitric oxide synthase (iNOS) in brain samples obtained from mice, we utilized specialized enzyme-linked immunosorbent assay (ELISA) kits, specifically Product No. SEA837Ra, sourced from USCN (USCN Business Co., Ltd., Wuhan, China). This analytical procedure was based on a colorimetric assay developed by Sigma-Aldrich (USA), which allows for highly accurate quantification of iNOS levels in the samples.

The process involves washing tissue slices with PBS, adding a tissue protein extraction reagent, homogenizing the mixture, centrifuging the samples, and collecting the resulting supernatants for analysis. The desired number of ELISA strip wells was prepared, and blank wells were left uncoated. Standards and samples were added to designated wells, and the 0 pg/mL well received 100 μL of Standard Diluent. The plates were incubated at 37 °C for 90 min, washed twice with wash buffer, and then incubated at 37 °C for 60 min. The plates were washed three times with wash buffer, and then 100 μL of the prepared biotinylated antibody was added to each well. The plates were then washed five times, and 100 μL of the prepared Enzyme Conjugate was added to each well. The plates were incubated at 37 °C in the dark, and the reaction was monitored visually and stopped once the color gradient was fully developed within 30 min. The plates were then mixed gently.

After conducting the assay, we measured the optical density (OD) of each sample at 450 nm using the Bio-Rad IMark Microplate Reader, equipped with version SW 1.04.02.E software. The OD measurements were then analyzed against a pre-established standard curve, which facilitated the conversion of these optical density values into measurable concentrations of iNOS. These concentrations were ultimately expressed in U/mg, ensuring clarity and uniformity for comparison across samples.

### Statistical analysis

The experiments’ results were presented as the mean value accompanied by the standard deviation (SD) for each group. A one-way analysis of variance (ANOVA) was conducted to assess the differences between the groups, followed by the Tukey *post hoc* test for further evaluation of the data. Statistical significance was determined based on a threshold P-value of 0.05 or lower for all comparisons made among the groups.

## Results

The phytochemical analysis performed using GC-MS unveiled a fascinating array of 18 distinct components within the JRLE, each characterized by unique peak areas and retention times, as outlined in **Table 1**. Among the noteworthy functional components, Resorcinol emerged prominently, showcasing its presence at a retention time of 13.635 min. Another intriguing compound identified was Juglone, recorded at 16.571 min, adding to the profile of bioactive substances present. Delving deeper into the analysis, Octadecanoic acid was detected at 23.410 min, followed closely by Squalene, which appeared at 28.945 min, both known for their beneficial properties. Additionally, the analysis highlighted the presence of Vitamin E at 32.838 min, a vital antioxidant, along with Stigmasterol, observed at 35.226 min, a compound recognized for its health-promoting effects. The analysis continued to reveal γ-Sitosterol at 36.426 min and Lupeol at 38.383 min, both of which contribute to the overall functional richness of the JRLE.

**Table 1:** Identification of phytochemical compounds by GC-Mass in JRLE.

t_R (min)_	Proposed compound	MW	Peak area	Peak area %	Formula
8.053	Carbamic acid, phenyl ester	137	715018	1.69	C_7_H_7_NO_2_
10.331	1-Butanol, 3-methyl-, formate	116	406313	0.96	C_6_H_12_O_2_
12.531	Phenol, 4-ethenyl-, acetate	162	190476	0.45	C_10_H_10_O_2_
13.635	Resorcinol	110	10109732	23.87	C_6_H_6_O_2_
14.530	9-Tetradecynoic acid, methyl ester	238	111405	0.26	C_15_H_26_O_2_
15.771	Phenanthrene, 9,10-dihydro-1-methyl-	194	502430	1.19	C_15_H_14_
16.571	Juglone	174	407699	0.96	C_10_H_6_O_3_
19.422	3-O-Methyl-d-glucose	194	15114032	35.69	C_7_H_14_O_6_
20.706	13-Heptadecyn-1-ol	252	373434	0.88	C_17_H_32_O
21.506	n-Hexadecanoic acid	256	1474337	3.48	C_16_H_32_O_2_
22.958	Phytol	296	984748	2.33	C_20_H_40_O
23.231	9,12,15-Octadecatrienoic acid, 2,3-dihydroxypropyl ester, (Z,Z,Z)-	352	1176676	2.78	C_21_H_36_O_4_
23.410	Octadecanoic acid	284	725328	1.71	C_18_H_36_O_2_
28.945	Squalene	410	1694195	4.00	C_30_H_50_
32.838	Vitamin E	430	4693186	11.08	C_29_H_50_O_2_
35.226	Stigmasterol	412	783745	1.85	C_29_H_48_O
36.426	γ-Sitosterol	414	1666724	3.94	C_29_H_50_O
38.383	Lupeol	426	1218915	2.88	C_30_H_50_O

During the infection induced by the *Pb*-parasite, the C57BL/6 mice displayed a striking array of neurological symptoms, characterized primarily by severe paralysis and pronounced ataxia. The findings presented in **Table 2** capture the intensity of parasitemia among the infected mice, revealing a notable average of 23.84 ± 2.06%. In evaluating plant extracts for infection mitigation, administering JRLE significantly reduced parasitemia in *Pb*-infected mice in a dose-dependent manner. Specifically, doses of 250 mg/kg and 500 mg/kg of JRLE reduced parasitemia by 13.25 ± 1.53% and 6.33 ± 1.18%, respectively. In marked contrast, the group of mice receiving chloroquine at a dosage of 10 mg/kg exhibited the most remarkable reduction in parasitemia, achieving an impressive level of just 3.11 ± 0.69%.

**Table 2:** Parasitemia level and CM signs for parasitized mice with *Pb* parasite.

Treatments	Dosage	Parasitemia level	Paralysis	Ataxia
Infected	–	23.84 ± 2.06	+++	+++
Methanolic extract of JRLE	250 mg/kg	13.25 ± 1.53	++	++
	500 mg/kg	6.33 ± 1.18	+	+
Chloroquine	10 mg/kg	3.11 ± 0.69	–	–

Data are presented as mean ± SD.

Note: absent (-), mild (+), moderate (++), severe (+++).

**Figure 2:**
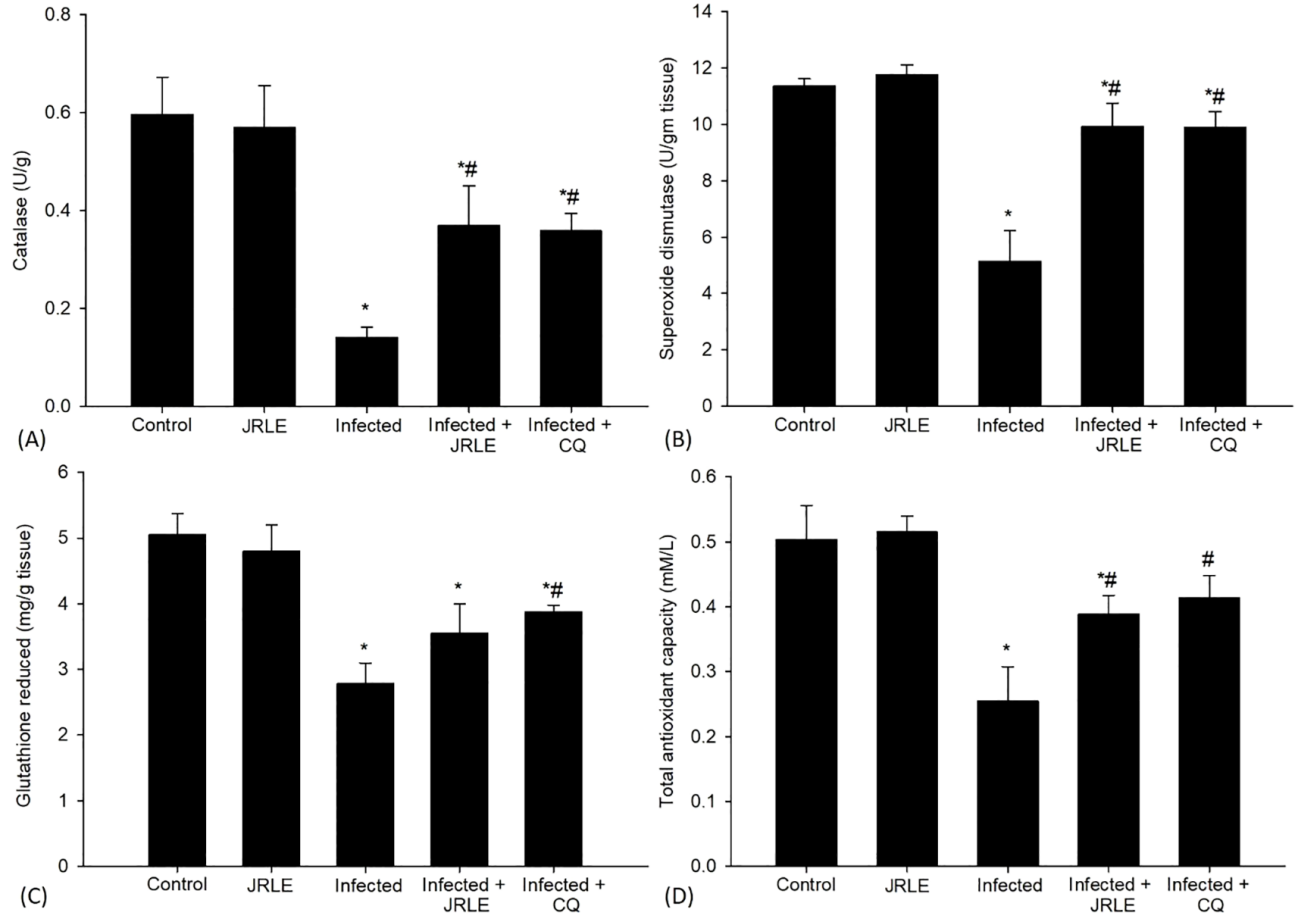
Bar graphs show four biomarkers: **(A)** Catalase, **(B)** Superoxide dismutase, **(C)** Glutathione reduced, and **(D)** Total antioxidant capacity. Each graph compares five groups: Control, JRLE, Infected, Infected + JRLE, and Infected + CQ. Infected groups generally show reduced levels, which are improved with JRLE and CQ treatment. * significance (p-value ≤ 0.05) against the control mice group, ^#^ significance (p-value ≤ 0.05) against the infected mice group.

Antioxidant enzymes such as CAT and SOD were analyzed 9^th^ day after infection (**Figure 2**). The CAT activity was markedly reduced in the brain tissue of the infected group, exhibiting a level of 0.14 ± 0.02 U/g, compared to the control group, which demonstrated a significantly higher CAT level of 0.59 ± 0.07 U/g. This substantial decrease suggests that the infection may adversely affect the enzymatic antioxidant defense mechanisms. In contrast, treatment with 500 mg/kg of JRLE and 10 mg/kg of CQ resulted in a notable restoration of CAT activity. Specifically, the CAT levels in the JRLE-treated group and CQ-treated group were measured at 0.37 ± 0.08 U/g and 0.36 ± 0.03 U/g, respectively. These findings indicate that both JRLE and CQ possess the potential to enhance CAT activity in the context of infection, as depicted in **Figure 2A**.

Furthermore, the analysis revealed a significant reduction in the levels of SOD in the infected group, where the SOD concentration dropped to 5.13 ± 1.10 U/gm, compared to a control group that maintained a baseline level of 11.36 ± 0.26 U/gm. This suggests that the infection negatively impacts the enzymatic activity of SOD, which plays a crucial role in combating oxidative stress. In contrast, treatment with JRLE and CQ led to a notable recovery in SOD levels among the treated mice. The SOD levels in the JRLE-treated group rose to 9.91 ± 0.84 U/gm, while the CQ group exhibited a similar increase with a SOD level of 9.89 ± 0.56 U/gm. These results indicate a potential protective effect of both treatments against the decline in antioxidant defense caused by the infection, as illustrated in **Figure 2B**.

**Figure 3:**
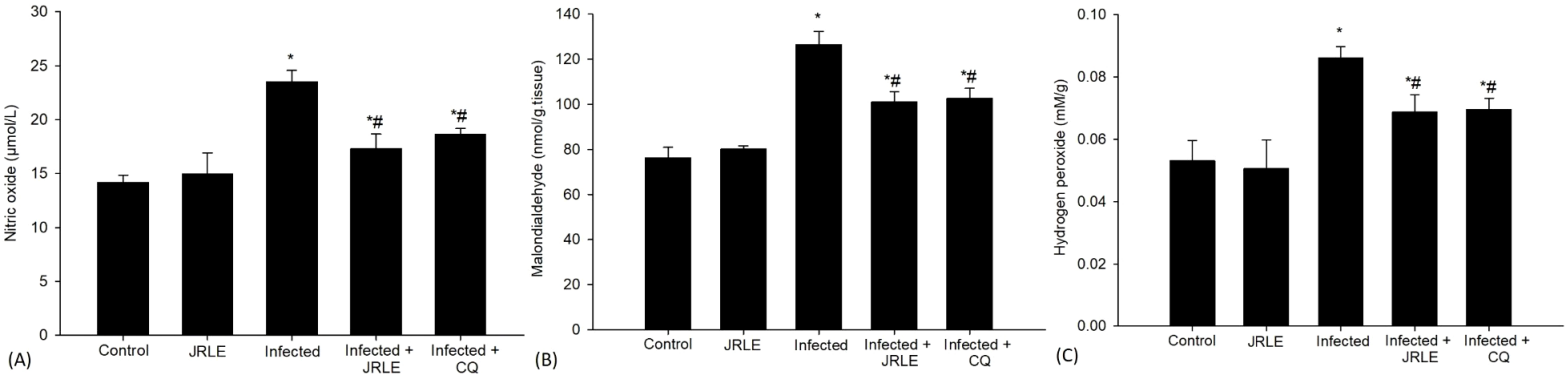
Bar graphs depict levels of **(A)** nitric oxide, **(B)** malondialdehyde, and **(C)** hydrogen peroxide across five groups: Control, JRLE, Infected, Infected with JRLE, and Infected with CQ. Nitric oxide and malondialdehyde levels increase in the Infected group, while both treatments reduce levels. Hydrogen peroxide shows a similar trend, peaking in the Infected group and decreasing with treatments. * significance (p-value ≤ 0.05) against the control mice group, ^#^ significance (p-value ≤ 0.05) against the infected mice group.

Antioxidant enzymes such as GSH, TAC, and NO were analyzed 9^th^ day after infection (**Figures 2** and **3**). The concentration of GSH, a critical antioxidant within the cells, was observed to be significantly decreased in the infected group, dropping from a baseline level of 5.05 ± 0.32 mg/g in the control group to just 2.78 ± 0.31 mg/g in the infected mice. This decline indicates a potential impairment in the antioxidant defense system of the mice due to infection. Following treatment interventions, the levels of GSH in the mice that received 500 mg/kg doses of JRLE and 10 mg/kg of CQ were notably elevated. Specifically, the GSH concentration increased to 3.55 ± 0.44 mg/g in the JRLE-treated group and reached 3.87 ± 0.10 mg/g in the CQ-treated group. These results suggest that both treatments were effective in upregulating GSH levels, restoring some degree of antioxidant capacity in the infected mice, as illustrated in **Figure 2C**.

Moreover, during the study investigating the effects of *Pb-*infection on antioxidant levels, it was observed that TAC significantly decreased in the infected group. Specifically, the TAC was measured at 0.50 ± 0.05 mM/L in the control group but dropped to 0.25 ± 0.05 mM/L in mice infected with the parasite (as illustrated in **Figure 2D**). This reduction indicates a compromised ability to neutralize free radicals, which can lead to oxidative stress and further complications during infection. Conversely, the administration of 500 mg/kg of JRLE and CQ resulted in a notable increase in TAC levels. Mice receiving JRLE (500 mg/kg) exhibited a TAC level of 0.39 ± 0.02 mM/L, while those treated with CQ reached a TAC of 0.41 ± 0.03 mM/L, suggesting that both treatments enhance the antioxidant capacity in these infected mice (**Figure 2D**). Furthermore, the DPPH assay, which evaluates the free radical scavenging ability of substances, revealed an impressive inhibition percentage of 90.50%, highlighting the potent antioxidant properties of the treatments applied.

In addition, the infection caused by *Pb-*parasite resulted in a notable surge in brain NO levels, with measurements rising to 23.51 ± 1.05 µmol/L. In contrast, the control group exhibited a significantly lower level of NO at 14.18 ± 0.68 µmol/L. This increase in NO is particularly relevant as it plays a crucial role in the termination of lipid peroxidation reactions, which are harmful processes that can lead to cell damage. Interestingly, the administration of a therapeutic dose of 500 mg/kg of JRLE, as well as 10 mg/kg of CQ, led to a significant decrease in NO levels. Following treatment, the NO levels were found to be reduced to 17.28 ± 1.38 µmol/L and 18.64 ± 0.51 µmol/L, respectively. This reduction suggests that both treatments may counteract the elevated levels of NO induced by the infection, potentially mitigating some of the oxidative stress and cellular damage associated with *Pb-*infection (**Figure 3A**).

Oxidative stress markers such as MDA and H_2_O_2_ were analyzed 9^th^ day after infection (**Figure 3**). The MDA levels, which serve as one of the definitive byproducts resulting from the peroxidation of polyunsaturated fatty acids within cellular structures, exhibited a noteworthy increase in the infected group. Specifically, MDA concentrations rose significantly from a baseline measurement of 76.27 ± 4.60 nmol/g in the control group to 126.49 ± 5.86 nmol/g in the group of mice that were infected. In contrast, when the infected mice were treated with JRLE or CQ, there was a marked reduction in MDA levels. The treatment with JRLE lowered the MDA concentration to 100.99 ± 4.58 nmol/g, while CQ brought it down to 102.51 ± 4.61 nmol/g. These results suggest that both JRLE and CQ were effective in mitigating the oxidative stress signified by elevated MDA levels in the infected group, as illustrated in **Figure 3B**.

Furthermore, in comparison to the control group, infection with *P. berghei* resulted in notable cellular damage, evidenced by a significant increase in the concentration of reactive oxygen species (ROS), particularly H_2_O_2_. The measured level of H_2_O_2_ in the infected group was found to be 0.08 ± 0.003 mM/g, indicating oxidative stress within the brain tissue (**Figure 3C**). To investigate potential therapeutic benefits, mice were treated with two different interventions: 500 mg/kg of JRLE and CQ. Both treatments demonstrated a remarkable efficacy in mitigating the elevated levels of H_2_O_2_ induced by *Pb-*infection. Following treatment, the concentrations of H_2_O_2_ were significantly reduced to 0.068 ± 0.005 mM/g for the JRLE group and 0.069 ± 0.003 mM/g for the CQ group, reflecting a successful alleviation of oxidative stress in the brain tissue (**Figure 3C**). This highlights the potential of these treatments to counteract the oxidative damage caused by parasitic infection.

**Figure 4:**
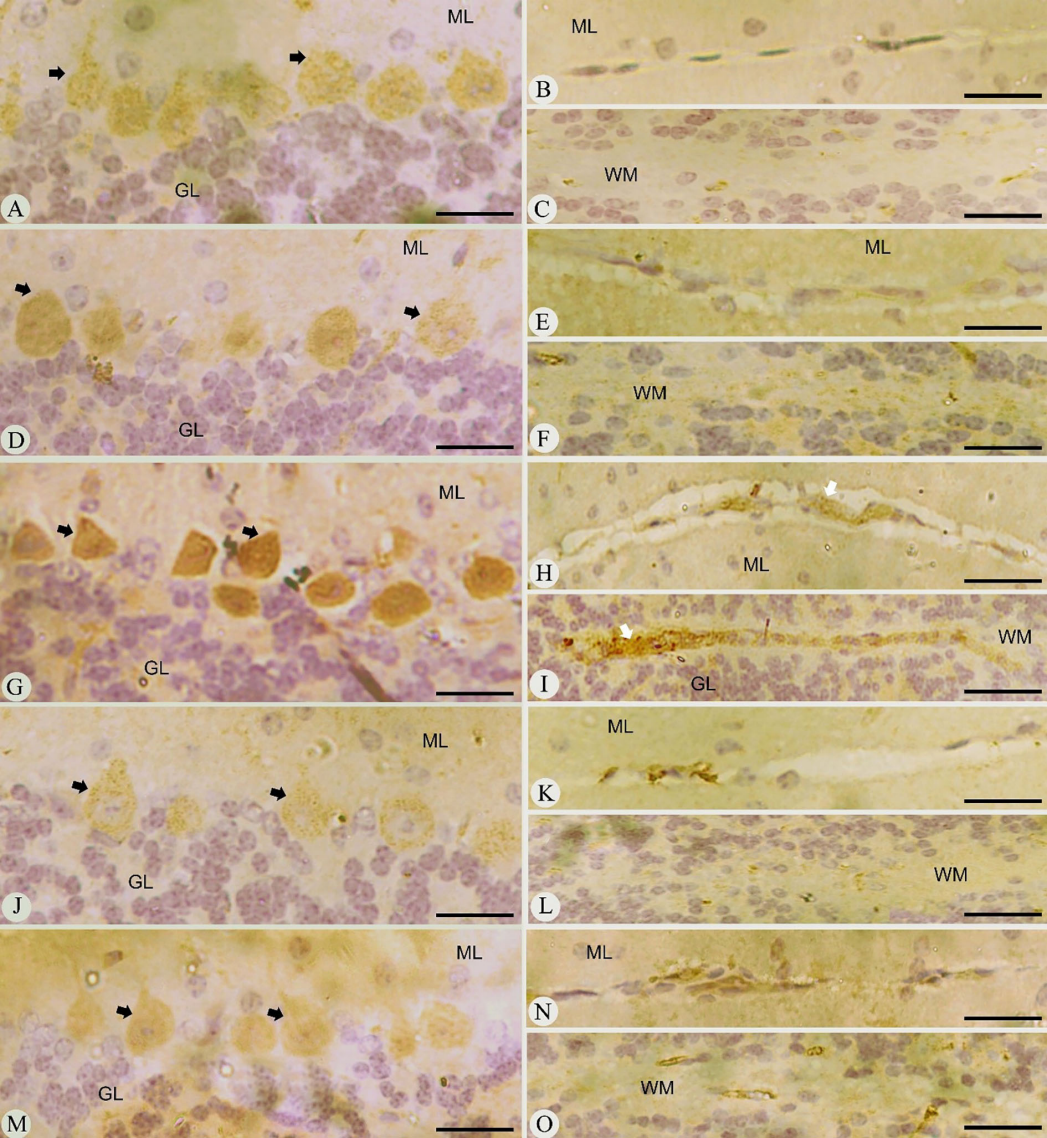
Immunohistochemical localization of iNOs in the brains of mice. **(A–C)** control mice group. **(D–F)** non-infected-treated mice group with 500 mg/kg JRLE. **(G–I)** *P. berghei* infected the brains of mice. **(J–L)** infected treated mouse group (500 mg/kg JRLE). **(M–O)** infected treated mouse group (10 mg/kg CQ). Note: ML, molecular layer; GL, granular layer; WM, white matter; Black arrows, Purkinje cell. Scale bar = 100µm.

Sections of brain tissue from various experimental groups were analyzed for the expression of iNOS, as illustrated in **Figure 4**. The results showed that *Plasmodium* infection led to a significant increase in iNOS expression levels, which were characterized by a high number of positive staining cells, mostly located around the Purkinje cell layer, as well as in both the cerebellar molecular layer and the white matter. This corresponds to a marked elevation in iNOS activity compared to the baseline levels observed in control mice that did not undergo *Pb*-infection. The upregulation of iNOS expression is thought to be triggered by immunological and inflammatory signals. Furthermore, the pathophysiological process of *Pb*-infection leads to an aberrant and spontaneous increase in NO production, which is linked to the altered expression of iNOS. Notably, post-treatment observations revealed significant alterations in iNOS expression levels in the infected-treated groups, specifically in both the JRLE-treated and CQ-treated groups, when compared to the infected group, as depicted in **Figure 4**.

**Figure 5:**
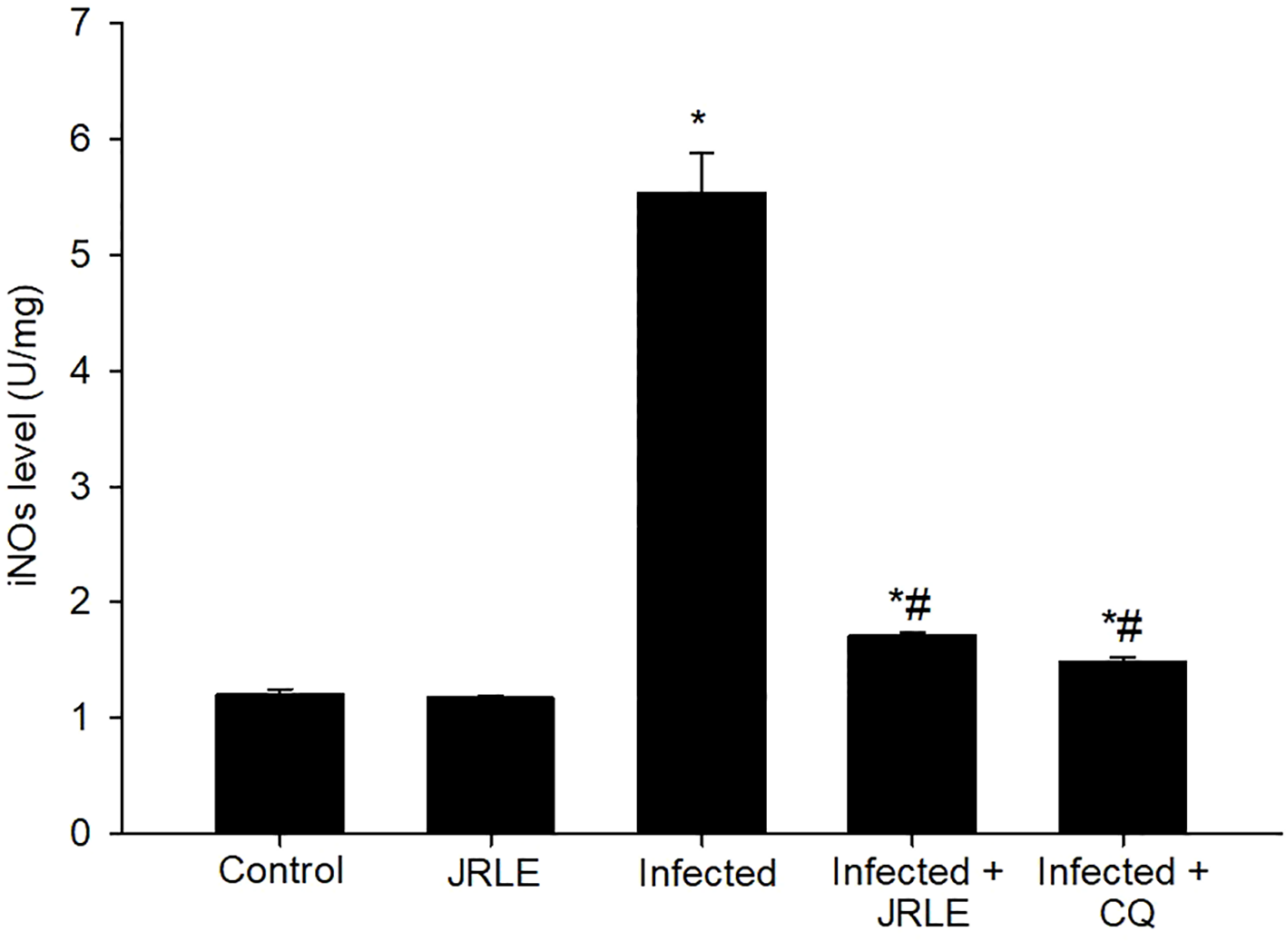
Bar graph showing inducible nitric oxide synthase (iNOS) levels in different groups: Control, JRLE, Infected, Infected + JRLE, and Infected + CQ. The Infected group has the highest iNOS level, 5.54 ± 0.34 U/mg, while treated groups with JRLE and CQ show lower levels of 1.71 ± 0.03 U/mg and 1.48 ± 0.04 U/mg, respectively. * significance (p-value ≤ 0.05) against the control mice group, ^#^ significance (p-value ≤ 0.05) against the infected mice group.

In our research, we delved deeper into the role of JRLE in the context of *Pb*-infection, particularly focusing on its influence on the production of iNOs, which is a crucial component of the body’s immune defense mechanism. To quantify iNOs levels, we employed ELISA methodologies, as illustrated in **Figure 5**. Our results revealed that infection with the *Pb*-parasite led to a markedly significant surge in the levels of iNOs, measuring at 5.54 ± 0.34 U/mg when compared to the control group. This elevation is indicative of the immune response triggered by the pathogen, as iNOs are synthesized primarily in response to various cytokines released during the infection process, highlighting their critical role in the host’s defense against parasitic attacks. Interestingly, treatment with JRLE demonstrated a remarkable efficacy in mitigating the elevated iNOs levels induced by the *Pb*-infection. The treated group showed a significantly reduced iNOs level of 1.71 ± 0.03 U/mg, which stands in contrast to the iNOs levels recorded in the infected group (**Figure 5**). This suggests that JRLE not only influences NO production but may also modulate the immune response to better manage the effects of *Pb*-infection.

## Discussion

Malaria remains a significant public health challenge and is one of the leading causes of illness and death, particularly in developing nations where healthcare resources may be scarce (Breman et al., 2004). The specific condition known as cerebral malaria (CM) is a severe complication resulting from the infection of humans with the *P. falciparum* parasite, while in experimental models, such as those using mice, the closely related *P. berghei* is commonly employed (Farahna et al., 2010). In recent years, there has been a growing interest in alternative treatments for malaria, particularly those involving herbal preparations that act as antioxidant agents. Several studies have documented the anti-malarial effects of various medicinal plants when tested against *P. berghei* infections in murine models (Hashim et al., 2024; Alharbi et al., 2025). In the current study, we aimed to evaluate both the anti-malarial and antioxidant properties of a specific herbal extract known as JRLE. The assessments were conducted using an *in vivo* model involving mice infected with chloroquine-sensitive strains of *P. berghei*, a common approach to gauge the effectiveness of new treatments against malaria. The research findings mirror those documented by Hearn et al. (2000), who identified a range of symptoms in mouse models suffering from CM. These symptoms often include respiratory distress, significant drops in body temperature, and neurological manifestations, such as ataxia, paralysis, and ultimately, coma, which can lead to death. Our observations corroborate this profile, further underlining the severity of the disease. Moreover, the anti-malarial efficacy of JRLE was notably demonstrated by a reduction in parasitemia levels among the treated mice. This therapeutic action can be attributed to the presence of various bioactive compounds within the JRLE, aligning with the data presented by Alharbi et al. (2025), highlighting the potential of herbal medicines as viable options in the fight against malaria.

Living organisms consistently produce reactive oxygen species (ROS) as a byproduct of cellular metabolism, a crucial process for maintaining physiological functions. However, various environmental factors and specific chemical applications can lead to an excessive accumulation of ROS within the body (Farombi et al., 2003). This overproduction of ROS plays a significant role as mediators of tissue injury, particularly during episodes of infection or in response to pharmacological interventions (Ojezele et al., 2017). In the context of malaria, this study highlights how malaria infection significantly disrupts the balance of oxidative stress markers and antioxidant defense mechanisms within the brain of the host. The resultant oxidative stress not only causes detrimental changes to red blood cells (RBCs) but also affects endothelial cells, which are critical for maintaining the integrity of blood-brain barriers (BBB). This disruption facilitates the penetration of the *Plasmodium* parasite into brain tissues, ultimately contributing to the pathogenesis of malaria and the development of serious neurological complications (Becker et al., 2004; Kumar and Bandyopadhyay, 2005). Additionally, the findings of this study indicate that treatment with JRLE can offer protective effects against oxidative stress in brain tissue during malaria infection. This protective role underscores the potential of JRLE not only to modulate oxidative damage but also to support antioxidant defense mechanisms in the face of malaria-induced stress. These observations are consistent with other recent findings reported by Alharbi et al. (2025), suggesting a broader relevance of these protective strategies in the context of malaria and oxidative stress.

The antioxidant enzymes, specifically superoxide dismutase (SOD) and catalase (CAT), are critical components in the body’s first line of defense against reactive oxygen species (ROS). These enzymes play essential roles in various physiological processes within cells, particularly when present in low concentrations, as they help maintain cellular homeostasis and prevent oxidative damage. However, when their concentrations become elevated, they can paradoxically contribute to cellular damage, adversely affecting proteins, lipids, and DNA structures (Birben et al., 2012). A notable observation in the context of *Plasmodium* infection is the marked reduction in SOD and CAT activity within the brain compared to control groups. This decrease has been documented in prior studies, emphasizing a consistent pattern wherein CAT activity diminishes significantly during infections caused by *P. berghei* and *P. vivax* (Seth et al., 1985; Erel et al., 1997; Mubaraki et al., 2017). The role of ROS, especially superoxide anions, has been demonstrated to inhibit CAT activity by facilitating the conversion of the enzyme into its inactive forms - ferryl (Fe^2+^) and ferroxy (Fe^3+^) states (Areekul and Boonme, 1987). This enzymatic alteration results in the accumulation of hydrogen peroxide (H_2_O_2_), which in turn exacerbates H_2_O_2_-induced oxidative stress, particularly within the context of *Plasmodium* infections (Aniya and Naito, 1983). Interestingly, treatment with JRLE has shown a significant enhancement in both SOD and CAT activity in the brains of subjects infected with *Plasmodium*. This improvement may be attributable to the presence of bioactive compounds such as squalene, stigmasterol, and lupeol within the JRLE. These compounds appear to possess properties that not only modulate oxidative stress in brain cells but also enhance neuronal communication, which is critical for maintaining brain function. This finding aligns well with the observations made in studies conducted by Bakrim et al. (2022); Park et al. (2023), and Zhang et al. (2023), suggesting that JRLE could be pivotal in mitigating oxidative stress effects in the context of *Plasmodium* infections.

In this study, it was observed that CM leads to a pronounced reduction in glutathione (GSH) activity, which is recognized as the primary antioxidant within the central nervous system (CNS). This finding aligns with the work of Vega-Rodríguez et al. (2015), who suggested that the depletion of GSH activity could be linked to the elevated levels of free radicals generated as a consequence of a high parasitic load from the *Plasmodium* species. In contrast, a noteworthy increase in GSH levels was recorded in the mice that were parasitized but subsequently treated with JRLE. This increase can be attributed to the high concentration of antioxidants found within the JRLE extract. The beneficial effects of these antioxidants are supported by findings from studies conducted by Turgut et al. (2006) and Zhao et al. (2018), which indicate that antioxidants play a crucial role in enhancing GSH levels in the context of parasitism. This suggests that antioxidant-rich treatments may provide a protective effect against oxidative stress induced by the parasitic infection, thereby helping to restore the balance of antioxidants in the body.

This study found that a particularly high antioxidant capacity is a characteristic of *Plasmodium* infection. This elevated antioxidant level results in an increase in TAC of the infected host. Such findings are consistent with studies by Gomes-Santos et al. (2011) and Sulistyaningsih et al. (2017), who found TAC to be a significant biomarker of disease across a range of pathophysiological conditions. Following treatment, the compound JRLE has proven to be an efficient inhibitor of the antioxidant enzymes that the malaria parasite produces after treatment. This inhibitory action plays a crucial role in reducing parasitemia, the presence of parasites in the blood. Furthermore, the extract’s antimalarial properties have been linked to its phenolic compounds. These compounds contribute to the antimalarial effect through several mechanisms: they elevate the oxidation levels within red blood cells (RBCs), disrupt the protein synthesis in the malaria parasite, and mitigate the oxidative damage caused by the parasite by neutralizing harmful free radicals. These findings are in line with the previous research conducted by Alharbi et al. (2025), further supporting the relevance of these biochemical interactions in combating malaria infection.

Nitric oxide (NO) is a crucial signaling molecule produced by activated macrophages, and it exhibits cytotoxic effects against a variety of pathogens, including the malaria-causing parasite *P. berghei* (Asensio et al., 1993; Sobolewski et al., 2005). Recent investigations have revealed that mice infected with *P. berghei* show significantly elevated levels of NO synthesis in their brains, suggesting a link between this small molecule and the host’s immunological response to parasitic infections. Previous studies, including those conducted by Nathan and Xie (1994) and Mubaraki et al. (2017), have highlighted the role of increased inducible nitric oxide synthase (iNOS) expression, which catalyzes excessive NO production, which may be regulated at many sites, including transcription, post-transcription, translation, and post-translational modification. Such elevated levels of NO are associated with various neurodegenerative diseases, indicating that NO has a dual role as both a defensive agent against pathogens and a potential contributor to neural damage. Interestingly, it has been noted that NO is involved in multiple physiological processes, particularly neurotransmission (Filho and Zilberstein, 2000). In the central nervous system (CNS), elevated NO levels can lead to the formation of peroxynitrite (ONOO-), a reactive nitrogen species that is implicated not only in the progression of brain lesions but also in the onset of neurological disorders associated with *P. berghei* infection (Martins et al., 2012; Yuste et al., 2015). Moreover, research by Maneerat et al. (2000) identified the expression of iNOS in various cell types within brain tissues—specifically in endothelial cells, neurons, astrocytes, and microglial cells—of subjects affected by cerebral malaria. This observation underscores the diverse cellular responses to inflammatory processes initiated by the infection. Further investigations revealed that treatment with JRLE is responsible for the effect of inhibition of the iNOS protein expression levels, suggesting the presence of bioactive components within JRLE that probably mediate at the level of arginine decarboxylase. This enzyme acts as an endogenous neuromodulator, which is induced in response to environmental stress and inflammation. The activation of arginine decarboxylase increases the expression of endothelial nitric oxide synthases (eNOS), while simultaneously irreversibly inhibiting neuronal nitric oxide synthases (nNOS) and downregulating the activity of iNOS, leading to a subsequent reduction in NO production. These findings align with studies conducted by Halaris and Plietz (2007), further reinforcing the intricate balance between NO synthesis and neuroinflammation in the context of cerebral malaria and other related neurological disorders.

Malondialdehyde (MDA) is recognized as a key biomarker for oxidative stress and is one of the most frequently investigated products resulting from lipid peroxidation. Within biological systems, cellular membrane lipids and proteins can undergo oxidative damage due to the highly reactive environment created by elevated levels of free radicals and ROS, as noted by Khan et al. (2015). In a recent study, it was observed that *Plasmodium* infection in mice led to a significant increase in brain MDA levels, accompanied by a notable reduction in glutathione (GSH) levels when compared to control mice. The elevated MDA concentrations in the brains of mice infected with *Plasmodium* suggest potential oxidative impairment of lipid and protein components that are essential for maintaining cellular membrane integrity. This oxidative stress likely contributes to an increased generation of ROS, exacerbating cellular damage and dysregulation. These findings are in line with previous research conducted by Hunt and Stocker (1990); Al-Shaebi et al. (2017), and Mubaraki et al. (2017), all of which explored the impacts of malaria on oxidative stress levels and redox status in biological systems. Importantly, the study also reported a significant reduction in MDA levels among parasitized mice that were treated with JRLE. This finding demonstrates the effectiveness of vitamin E and juglone, which have been identified as bioactive components within JRLE, in reducing oxidative stress. These substances seem to have characteristics that efficiently reduce or scavenge the ROS generated as a result of the host’s immunological response during the *Plasmodium* infection. The findings of this study support the possible use of antioxidant treatments in the management of oxidative damage linked to malaria and are consistent with subsequent research by Rychter et al. (2022) and Cintesun et al. (2023). Overall, this study emphasizes the complex interplay between malaria infection, oxidative stress physiology, and the therapeutic potential of natural compounds in mitigating oxidative damage.

## Conclusion

The accumulated evidence suggests that administering extracts from the leaves of *J. regia* to mice infected with *P. berghei* may help alleviate oxidative stress. Both the management and prevention of malaria infections seem to benefit greatly from this treatment. Further studies should be conducted to isolate the bioactive component of the extract that is responsible for the observed antiplasmodial and antioxidant activities for the development of a new therapeutic agent.

## Data Availability

This published article includes all the datasets generated or analyzed during this study. Requests to access the datasets should be directed to rabdelgaber@ksu.edu.sa.
